# Risk Factors for Transition of Care in Disorders of Gut–Brain Interaction: A Narrative Review and Expert Opinion

**DOI:** 10.3390/children12091209

**Published:** 2025-09-10

**Authors:** Miguel Saps, Samantha Arrizabalo, Jose M. Garza

**Affiliations:** 1 Department of Pediatrics, Division of Gastroenterology, Hepatology and Nutrition, Miller School of Medicine, University of Miami, Miami, FL 33136, USA; msaps@med.miami.edu; 2 Children’s Center for Digestive Health Care, Children’s Healthcare of Atlanta, Atlanta, GA 30342, USA; jgarza@gicareforkids.com

**Keywords:** children, adolescent, adulthood, transition of care, program

## Abstract

Background: Disorders of gut–brain interaction (DGBI) have a significant impact on the quality of life of children and families. Forty percent of children with recurrent abdominal pain continue to have symptoms into adulthood. Specialized programs for the transition of adolescents with DGBI to adult care are scarce. There are no widely accepted guidelines for transition of care. Identifying risk factors for persistence of symptoms into adulthood is key to identifying the optimal population that should be part of such programs and guidelines design. Methods: A narrative comprehensive review was conducted using predefined keywords to identify risk factors for persistent DGBI in children/adolescents. Results: Female sex, psychological distress, family history of DGBI, and certain comorbidities had stronger evidence for persistence, whereas other risk factors rely on limited data. Conclusions: It is suggested that transition programs should focus on adolescents presenting with multiple coexisting risk factors. The program should at least include pediatric and adult neurogastroenterologists, dieticians, psychologists, and social workers. Tertiary prevention through psychological support, school-based programs, and management of anxiety and sleep disturbances may reduce the persistence of symptoms. Prospective studies should refine risk stratification and guide transition strategies.

## 1. Introduction

Thirty to forty percent of community children report abdominal pain [1]. Children with abdominal pain have a poor quality of life [2] and frequent comorbidities such as headaches, sleep problems, anxiety, and depression, among others [3]. Only a small proportion of these children seek medical care for their symptoms [2]. Children who consult for abdominal pain frequently have long-lasting symptoms well into adulthood. Approximately 40% of children with recurrent abdominal pain (RAP) and 30% of those who meet Rome IV criteria for an abdominal pain-predominant DGBI continue to experience abdominal pain at 24 years [4]. Sixty percent of them have irritable bowel syndrome (IBS) and functional dyspepsia [4].

Children and adults with DGBI impose a significant burden on the healthcare system [5]. Adults with IBS are less likely to succeed professionally, which also adds to the personal and societal burden [6]. Despite the magnitude of the problem, research on the risk and protective factors involved in the persistence of symptoms into adulthood remains scarce. As a result, there is little data to inform strategies for adolescents with long-term symptoms based on evidence [7,8].

Few institutions have programs for transition of care for adolescents and young adults with DGBI, and there are no universally accepted guidelines on how to design these programs [9]. The importance of such programs was highlighted by multiple pediatric and adult societies, and efforts have been made to outline certain aspects of the programs [10,11]. A common problem of all these initiatives is that they are based on information from existing guidelines on organic diseases [12,13]. In contrast to transition programs for patients with liver failure [14], cystic fibrosis [15], inflammatory bowel disease [16], and celiac disease [17], which are the bulk of the transition guidelines, children with DGBI frequently improve [18]. Therefore, recommendations developed for lifelong diseases may not be fully applicable to children with DGBI, as only a minority will continue to experience symptoms into adulthood. Consequently, transition planning at an early age, as is often recommended for organic diseases, may not be necessary for most adolescents with DGBI. Moreover, early inclusion into a transition program can send a defeating message to the patient, becoming a self-fulfilling prophecy of poor outcomes [19]. A positive message and outlook are key to the successful management of patients with DGBI [20,21]. Children with DGBI can benefit from the important placebo effect shown in some studies, which can help heal, while a message of likely continuation with symptoms may have a nocebo effect that compromises the outcome [22]. Based on all these factors, it is key to establish the target population for a successful transition program. [4,23,24]. We provide a narrative overview of articles on the persistence of symptoms into adulthood, selected for relevance [4]. The following recommendations for transition of care are based on the evidence drawn from these publications in combination with personal expert opinion. We embark on this effort knowing that in no way will we be able to provide definitive recommendations, and those will likely need to be modified as the field evolves and new data are published.

## 2. Materials and Methods

A narrative comprehensive search of the PubMed database was conducted for studies published between January 1991 and March 2025. Keywords included: “sex,” “family history of gastrointestinal symptoms,” “food hypersensitivity,” “asthma and eczema,” “antibiotic use,” “anxiety”, “depression,” “eating disorders,” “poor quality of life,” “adverse events,” “abuse,” neglect,” “sleep,” “comorbid somatic symptoms,” “acute gastroenteritis,” “celiac disease,” “inflammatory bowel disease,” and “down syndrome,” “attention-deficit/hyperactivity disorder,” “autism,” “postural orthostatic tachycardia syndrome” using “AND” “disorders of gut-brain interaction” or “recurrent abdominal pain” or “irritable bowel syndrome (IBS)” in combination with “children” using “AND”/“OR” operators as appropriate. We included observational studies, randomized controlled trials, and systematic reviews involving children and young adults (ages 0–24 years) published in English. Animal studies and case reports were excluded. Study selection was performed by Miguel Saps (M.S.), and only articles meeting these criteria were considered for inclusion, resulting in a total of 72 articles reviewed.

## 3. Results

The reviewed studies identified psychological, biological, and social factors that may influence gut–brain interaction and contribute to the persistence of DGBI symptoms into adulthood. These factors are summarized in Table 1.

- **Biological factors**

**Early life events**

There is conflicting data on the possible effects of birth-related factors such as prematurity, low birth weight, type of delivery, and duration of breastfeeding. **Prematurity and low birth weight—**premature infants are more likely to undergo invasive maneuvers, surgeries, infections, use of antibiotics, and maternal separation. However, most of these factors have not been clearly linked to long-term pain [25]. In fact, a study by Olén et al. (2018) [26] found preterm birth to be protective for IBS in adults. Low birth weight has been associated with a slightly increased risk of IBS (1.11; CI 1.01–1.22), a finding replicated in a Norwegian twin study [27]. In contrast, a large Swedish study found no relationship between preterm birth or birth weight and IBS at 24 years of age [28]. **Mode of delivery—**US and Colombia’s cohort studies found no association between cesarean delivery, use of antibiotics, or maternal age with IBS in adult subjects [29,30]. These results contradict a Scandinavian cohort study by Waehrens et al. [31], which showed a significant association between cesarean delivery and IBS at 18 years of age. Given the small effect size and inconsistent findings, the mode of delivery is unlikely to serve as a reliable basis for targeting adolescents for transition programs. **Breastfeeding—**While breastfeeding has clear benefits for nutrition, bonding, and immunity, there is no clear evidence that children who were breastfed for longer periods of time have a lower risk of developing DGBI as adults [7,28]. Thus, there is no apparent benefit of targeting children for transition programs based on breastfeeding history alone.

**Sex—**Female sex has been consistently associated with abdominal pain and IBS in children and adults [32], and with the persistence of RAP and IBS from childhood into adulthood [33]. This may be explained by increased healthcare-seeking behavior [34], a higher prevalence of comorbidities such as fibromyalgia [35] and migraine with aura [36], and differences in coping mechanisms and stress responses [37].

**Parental history of gastrointestinal (GI) symptoms—**multiple studies have shown that a history of GI problems in parents is a risk factor for the development and persistence of symptoms. A birth cohort study found that parental IBS is the strongest predictor of symptom persistence from ages 16 to 24 [28]. Although the mechanisms underlying this association are not yet completely understood, maladaptive coping and social learning have been associated with poor patient outcomes [38,39].

**Allergic diseases—**Children with asthma and eczema are more likely to have IBS and RAP as young adults [40]. Infants with **cow’s milk protein allergy** have an increased risk of abdominal pain by age 8 [41]. A similar finding was reported in children with allergic proctocolitis, who had a four-fold risk of developing abdominal pain–predominant DGBI [42]. However, there are no studies following this group of children into adulthood. A study has shown that **food hypersensitivity** has also been linked to IBS in young adults; however, this association was based on self-reported survey data, making it unclear whether the mechanism involves an allergic reaction [40]. Therefore, it is not possible to distinguish the reaction from visceral hypersensitivity or other mechanisms.

**Infant colic—**Evidence linking infant colic with abdominal pain later in childhood is conflicting. Some studies report an association with childhood abdominal pain [43,44,45,46], while others do not [4,47]. Notably, no studies to date have demonstrated a link between infant colic and the persistence of abdominal pain into adulthood.

**Abdominal pain during childhood and adolescence—**Abdominal pain at 12 and 16 years is associated with RAP and IBS at 24 years of age [28]. Approximately one-third of 16-year-olds with IBS meet the diagnosis at 24 years of age, and another 10% report abdominal pain without meeting criteria for IBS [28].

**Celiac disease and inflammatory bowel disease—**A subset of children and adults with celiac disease and inflammatory bowel disease (IBD) have overlapping IBS [48,49]. However, there have been no pediatric studies that investigated the long-term prognosis of these children.

**Neurodevelopmental disorders—Attention deficit hyperactivity disorder and autism** have been associated with a higher likelihood of abdominal pain disorders [50,51,52]. However, there are no published studies demonstrating that these conditions make children more likely to manifest abdominal pain as adults. **Down syndrome—**Similarly to the previous diseases, Down syndrome has been linked to DGBI (mostly defecation disorders) in children [51,52]. Children with both Down syndrome and autism have a higher prevalence of DGBI [51,53,54,55] but no studies have examined whether these conditions are risk factors for the persistence of RAP or abdominal pain DGBI into adulthood.

**Postural orthostatic tachycardia syndrome (POTS)** is more common in young women and is frequently associated with comorbidities [56]. Nausea, abdominal pain, constipation, and bloating are often reported in children and adults with POTS [57,58]. However, there are no follow-up studies on the persistence of GI symptoms in children with POTS into adulthood. Given the significant burden experienced by children with POTS and IBS, it would be reasonable to consider them as part of transition-of-care planning, even in the absence of long-term outcome data.

- **Psychological factors**

**Psychological distress and quality of life—**Children with abdominal pain are more likely to have anxiety, depression, and poor quality of life [23]. Data from a community study showed that the presence of anxiety and depression and poor health-related quality of life at 16 years of age was associated with RAP and IBS at 24 years [28]. A study conducted among consulting children with abdominal pain found that this group of children is also more likely to have anxiety during childhood that persists into early adulthood [33].

Clinic-based studies have identified three trajectories of abdominal pain symptoms. Children with a greater number of psychological comorbidities, including anxiety and depression, are more likely to continue experiencing abdominal pain years later [59,60].

**Eating disorders—**Children with DGBI are at higher risk for eating disorders, orthorexia, and avoidant/restrictive food intake disorder (ARFID). The Avon longitudinal study of parents and children found that RAP at 7 and 9 years was associated with increased risk of fasting for weight control at 16 years of age [61]. Children with eating disorders are also more frequently diagnosed with DGBI and dysmotility [62,63]. The use of social media, in particular some apps, has been associated with a higher risk of eating disorders [64,65]. In addition, recent evidence suggests that social media itself may influence children with DGBI, highlighting a potential link between online behaviors and gastrointestinal symptoms [66]. However, no published studies have examined whether eating disorders predispose to the persistence of abdominal pain into adulthood.

**Sleep disorders—**In adults, poor sleep quality has been shown to influence next-day GI symptoms [67]. Children with abdominal pain are more likely to suffer from sleep disorders [32,68]. Furthermore, short sleep duration at age 16 has been associated with abdominal pain at 24 years of age [28].

- **Social and environmental factors**

**Smoking during pregnancy and secondhand smoke in childhood—**Parental smoking is a risk factor for abdominal pain at ages 12 and 16 [28]; however, studies have not shown an association between maternal smoking during pregnancy and long-term abdominal pain [69].

**Abuse—**Multiple studies have shown an association between various types of abuse during childhood and long-term abdominal pain and other somatic symptoms in adulthood [70,71]. Abuse is also linked to psychological distress, including anxiety and depression [72], which are themselves risk factors for persistent RAP and IBS into adulthood. Family violence, hate crime, emotional, sexual, and physical abuse, as well as neglect, are associated with adult mental health problems that, in turn, are linked to IBS [73,74]. Bullying, a specific form of abuse, has been associated with an increased prevalence of abdominal pain and other somatic complaints [75,76,77,78]. However, no studies have examined the long-term relationship between childhood bullying and IBS in adulthood.

**Infections—Urinary tract infections—**Hospital-based and national insurance database studies in Taiwan and the United States have shown an association between urinary infections during the first 2 years of life and IBS at 12 years of age in hospital-based cohort studies [79,80]. Although antibiotic exposure during UTI episodes may act as a confounding factor contributing to this association, additional mechanisms have also been proposed. In particular, neural bidirectional cross-organ sensitization may help explain how pathological changes in the urinary tract can result in long-term increased pain sensitivity in the gastrointestinal system [81]. However, no studies have investigated whether this association persists into adulthood. **Acute gastroenteritis—**Acute gastroenteritis, particularly of bacterial origin, is the most clearly identifiable trigger of IBS in both children and adults. Approximately one in ten adults develops IBS following an acute gastrointestinal infection [82,83]. Functional dyspepsia [7] and functional constipation [84] have also been reported after such infections. Associations have been documented with a range of pathogens, both in outbreak settings [85,86,87] and isolated cases [88,89]. Viral infections are thought to have short-lived effects, making persistence into adulthood unlikely [90]. Six percent of community children with acute gastroenteritis (likely of viral origin) have new onset IBS at 6 months [89], while another study did not find an association between rotavirus gastroenteritis and abdominal pain at 6 months [90]. In contrast, bacterial and parasitic infections can have long-lasting consequences. In an Italian school outbreak of *Salmonella*, children were more likely than adults to report persistent symptoms at 16-year follow-up [91]. An *E. coli* outbreak in Walkerton, Ontario, led to gastrointestinal symptoms persisting two to three years in affected children [92], while outbreaks of *Cryptosporidium* in Sweden [85] and Milwaukee [93] continued to report abdominal pain and diarrhea, persisting up to 5 years post-infection. Although there are studies in adults investigating the risk factors that predispose to the development of post-infection irritable bowel syndrome in adults including female sex [82], younger age, psychological distress [94], and severity of the acute episode [89], very few studies have investigated these risk factors in children, and none have assessed their impact on persistence into adulthood.

**Antibiotics—**Studies have shown an association between the use of antibiotics and the incidence of RAP and IBS in children and adults, as well as the persistence of IBS from adolescence into young adulthood [95,96]. Higher cumulative antibiotic exposure has been linked to an increased likelihood of persistent GI symptoms into adulthood [28]. Whether antibiotics are an independent risk factor or merely a proxy for the underlying illnesses prompting their use remains unclear [28]. However, the fact that this association has been shown consistently with all types of antibiotics studied suggests that antibiotics per se may be an independent risk factor, probably through changes in the microbiome [69].

**Figure 1:**
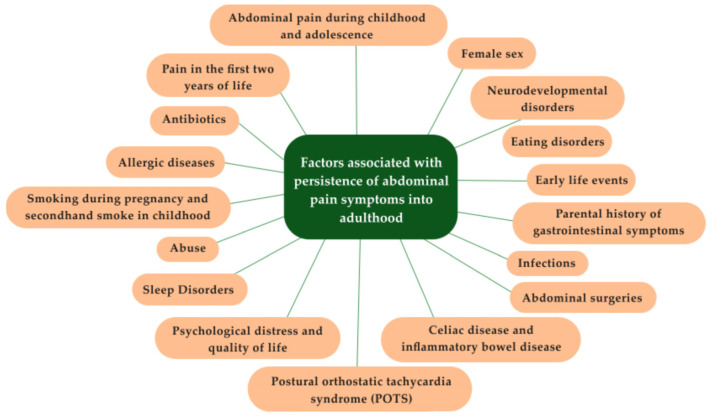
Factors associated with persistence of abdominal pain symptoms into adulthood.

Figure 1 summarizes the risk factors for the persistence of GI symptoms into adulthood based on evidence when available and expert opinion when data are insufficient.

## 4. Discussion

**Identification of population for transition of care—**The high number of adolescents with abdominal pain, the complex logistics of running a transition of care program, the cost associated with such a program [97,98], and the fact that approximately two-thirds of the children will improve before adult years [24,99], makes enrolling every adolescent with abdominal pain into a transition program unnecessary, impossible, and inconvenient. Thus, the challenge lies in how to select the group of patients who are more likely to benefit from the program.

The aforementioned data can provide an approximation of who should be the target population for a transition-of-care program based on differences in risk of persistence of abdominal pain (including IBS) into adulthood. The analysis of the data can also help inform about key healthcare members who should integrate the program and help draw recommendations on some novel elements that are frequently not considered.

Among the many factors that have been found associated with long-term abdominal pain, only a few have been shown to constitute risk factors for persistence into adult years. The sparsity of data, combined with methodological limitations and the absence of data replicating most studies, preclude us from providing strong recommendations on which patients should be part of a transition of care. In the absence of these data, in the opinion of the authors, it is suggested that the various factors have different weights, and it is more likely that the combination of factors predisposes to the persistence of symptoms into young adulthood. Available studies suggest that factors such as female sex, anxiety and depression, poor quality of life, eating disorders, POTS, IBD, family history of GI symptoms, abuse, and sleep problems may be associated with persistence, although evidence remains limited and largely observational. However, it remains unclear whether each factor is an independent predictor or reflects the interplay of multiple other variables. Future research should confirm the individual role of each factor in the persistence of symptoms. Until large, well-designed prospective studies lead to a definitive conclusion of what factors should inform the transition of an adolescent with DGBI to adult care, the decision should be made on a case-by-case basis in consultation with the patient and caregivers.

**Figure 2:**
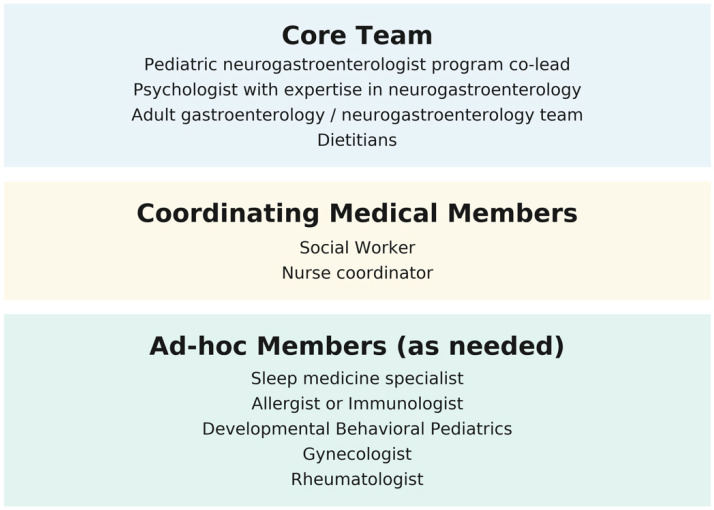
Core, ad-hoc, and coordinating members of the transition of care team.

**Members of the team—**Each of the risk factors is associated with its own set of comorbidities, making the care of these children inherently complex. The transition of care team needs to be interdisciplinary, well-coordinated, and guided by shared goals, principles, and approaches. Many of the identified risk factors are linked to psychological disorders, reinforcing the importance of incorporating the biopsychosocial model as the framework for abdominal pain disorders. **Psychologists** with expertise in neurogastroenterology should be a key part of the program. Psychological interventions such as cognitive behavioral therapy (CBT) [100] and hypnotherapy [101,102] have demonstrated efficacy, both in person and through online delivery [103]. The psychologist, in consultation with the family, should determine the most appropriate therapy type and delivery method. Some of the psychological interventions may be focused on the family and may include addressing maladaptive coping skills [104]. Although most of the studies have been conducted on CBT and hypnotherapy, other techniques may be recommended in individual cases. As an example, eye movement desensitization and reprocessing (EMDR) may be of value in the case of patients with a history of trauma [105]. Given that children with abdominal pain often experience high school absenteeism [106], functional disabilities, and poor quality of life [107], the participation of **social workers** is of utmost importance. They can assess the patient’s social environment, help design and instrument specific directives of care, and communicate with teachers, school authorities, and agencies. The **pediatric neurogastroenterologist** has an important role in evaluating and managing possible underlying GI conditions while reinforcing the biopsychosocial model in collaboration with psychologists. Both pediatric neurogastroenterologists and psychologists should lead the program, while the entire team should participate in the discussion and treatment decisions. Plans of care should be tailored to each case and the adolescent’s individual circumstances. Therefore, the team should include **ad-hoc members** when specific needs arise, as in the case of sleep problems, severe headaches, fibromyalgia, autonomic dysfunction, developmental problems, allergies, and gynecological problems. Pain medicine and rehabilitation specialists should be part of the team, although their role and need may vary on a case-by-case basis. The program should be integrated by a **nurse coordinator** who will ensure the plans of care are implemented, coordinating services, and maintaining communication with families and providers. Finally, at the point of transition, plans should be discussed and coordinated with the **adult gastroenterology team, and when possible, with a neurogastroenterology program. Figure 2** outlines the proposed structure of the transition care team, including core members, ad-hoc members, and coordinating medical members.

**Age of inclusion into the program—**The program should be focused on adolescents. A study has found that 16-year-old adolescents are more likely to have persistent abdominal pain into adulthood than younger children [28]. Thus, in contrast to transition programs for organic diseases that propose early participation, expert opinion suggests that 16 years may be an appropriate age for inclusion in the program. The age of inclusion may differ depending on the characteristics of the local healthcare system and individual circumstances. Future studies should compare outcomes of the various ages of enrollment into the transition program.

**Tertiary prevention—**The Neurogastroenterology Program will also serve as a tertiary prevention initiative [108]. Unlike organic diseases, where symptoms will always persist, most children with DGBI improve over time, making this type of transition program unique. Improving some of the actionable risk factors could not only help prevent the progression of GI symptoms but also improve the quality of life and daily function of patients and families.

The progression of symptoms is not limited to consulting patients. To maximize the impact of the program, tertiary prevention should transcend the medical facilities to include children in schools. The analysis of the risk factors listed in Table 1 suggests that some of those are amenable to school-focused interventions. Non-pharmacological approaches in the management of abdominal pain, such as physical exercise, yoga, mind-body therapy, and others, may help improve quality of life and could potentially refocus attention away from pain, although evidence in pediatric populations remains limited [109,110]. A detailed description of all possible school or home-based interventions is beyond the scope of this publication.

## 5. Conclusions

Available evidence on risk factors for persistent DGBI into adulthood remains limited. Prospective studies are needed to clarify the relative weight and interplay of these factors. The recommendations presented in this review are based on expert opinion informed by the available data and are intended to inform clinical practice until more robust evidence is available. Transition programs for DGBI are unique, as not all adolescents will require transfer to adult care; a positive, individualized approach focused on tertiary prevention may help reduce symptom persistence and improve quality of life.

## Figures and Tables

**Table 1:** Biopsychosocial classification of risk factors for persistent DGBI.

Biological Factors	Psychological Factors	Social and Environmental Factors
- Early life events - Female sex - Parental history of gastrointestinal symptoms - Allergic diseases - Pain in the first two years of life - Abdominal pain during childhood and adolescence - Celiac disease and inflammatory bowel disease - Neurodevelopmental disorders - Postural orthostatic tachycardia syndrome	- Psychological distress and quality of life - Eating disorders - Sleep disorders	- Smoking during pregnancy and secondhand smoke in childhood - Abuse - Infections - Antibiotics - Abdominal surgeries

## Data Availability

Not applicable.

## Abbreviations

The following abbreviations are used in this manuscript:

DGBI	Disorder of gut–brain interaction
RAP	Recurrent abdominal pain
IBS	Irritable bowel syndrome
GI	Gastrointestinal
ARFID	Avoidant/restrictive food intake disorder
IBD	Irritable bowel syndrome
POTS	Postural orthostatic tachycardia syndrome
CBT	Cognitive behavioral therapy
EMDR	Eye movement desensitization and reprocessing
